# Social Media-Based Collaborative Learning Effects on Student Performance/Learner Performance With Moderating Role of Academic Self-Efficacy

**DOI:** 10.3389/fpsyg.2022.903919

**Published:** 2022-07-08

**Authors:** Shuai Liu, Ghulam Hussain Khan Zaigham, Rao Muhammad Rashid, Ahmad Bilal

**Affiliations:** ^1^School of Marxism, Dalian Maritime University, Dalian, China; ^2^Department of Management Sciences, COMSATS University Islamabad, Islamabad, Pakistan; ^3^Department of Management Studies, Bahria University Karachi Campus, Karachi, Pakistan; ^4^Department of Computer Science, Sir Syed University of Engineering and Technology, Karachi, Pakistan

**Keywords:** social media, collaborative learning, academic self-efficacy, perceived ease of use, learning performance (LP)

## Abstract

Social media has always been described as the channel through which knowledge is transmitted between communities, students, and learners. This social media has been utilized by university students in a way to encourage collaborative learning and social interaction. This study explores the use of social media in the process of collaborative learning among university students in China using a survey method, a total of 583 students from different universities were surveyed in this study. Through this investigation, different factors enhancing collaborative learning among university students in the context of using social media are going to be examined. Structural equation modeling (SEM) and hierarchical regression were used to analyze the suggested hypothesis. Results show that perceived benefit, active learning, and interaction with students are significantly related to social media collaboration. However, perceived ease of use and perceived usefulness have an insignificant effect on social media collaborative learning. Additionally, students’ academic self-efficacy significantly moderates the relationship between social media collaboration and learning performance. The implication and limitations of the study are also discussed in the last section.

## Introduction

A learner’s attitude is vital with respect to collaborative learning, and the result of this process depends upon the learner’s attitude toward social media-based collaborative learning (SMBCL); usually, a student’s attitude toward collaborative learning is positive. According to Liao et al. (2015) social media-based “collaborative learning” is a progression in which students learn together, and promotes learning through social interactions and teamwork. Korkmaz (2012) uses the term “collaborative learning” to refer to a process in which two or more people work together to adopt a group learning attitude and improve their learning from the existing group or team. Chapman and Van Auken (2001) discussed the faculty member or instructor’s role in group management and said that instructor has a significant positive effect on student learning attitude in teamwork. Pfaff and Huddleston (2003) found student assignments, project work, project grade, evaluation, and ability to work in teams improve from social media-based collaborative learning. Fu et al. (2009) showed that information technology and social media-based websites have designed a supportive and friendly collaborative learning environment for student motivation and engagement. According to Junco (2012), social media has become a tool for collaborative learning, and faculty members give extensive attention to the collaborative learning skills of students. Many researchers such as Mao (2014) in their previous studies focused on the positive and negative attitudes of the student toward social media based educational learning and explored that student opinions regarding educational use of social media are usually good, that they make learning enjoyable, motivating, collaborative, and that they increase their enthusiasm for teamwork. However, other researchers like Lim and Richardson (2016) suggested that social media has a detrimental impact on many students’ views and that they believe social media does not help them accomplish academic goals. Paliktzoglou and Suhonen (2014) investigated student-specific opinions regarding using social media as a problem-solving aid in learning.

Moreover, students appear to have a favorable learning attitude toward social media-based learning but prefer a direct connection with other teachers and students (Morreale et al., 2015). Korkmaz (2012) stated that students’ attitudes play an essential role in collaborative learning and online learning. Alkhathlan and Al-Daraiseh (2017) investigated that many aspects influence attitudes toward using social media-based collaborative learning, including collective efficacy, usefulness, playfulness, and personal innovativeness. Akçayır and Akçayır (2016) investigated that there is still a lack of empirical research related to the previous attitudes of students toward social media-based collaborative learning. This research aims to examine prior students’ attitudes regarding social media-based collaborative learning and the impact of faculty members on their learning performance.

This research aims to assess the effect of the following variables: learning performance of students toward social media-based learning, perceived usefulness, perceived ease of use, academic self-efficacy, active learning, and student interaction with faculty and learner performance. This study is based on survey data. This study has several theoretical and practical implications. Firstly, this article offers an empirical investigation of graduate students working with social media-based collaborative teamwork throughout a semester of financial management at Chinese institutions. Secondly, this study was conducted in China, Chinese students use several social media tools for learning purposes. Thirdly, this study also investigated the moderating role of academic self-efficacy in the relationship between social media collaboration and learning performance.

## Literature Review

Learning is a continuous process, and every individual improves their learning over time from the latest mode of learning. According to Molinillo et al. (2018), teachers should aim to instill a better learning attitude in their pupils, diminishing the risk associated with a lack of face-to-face interaction with their peers and reducing the fear of failing to complete assigned tasks. This study is based on concepts from social cognitive theory (SCT), social comparison theory, and the technology acceptance model (TAM). As a result, this research does not evaluate these ideas. Still, it does highlight a number of their methods that may influence student views regarding social media-based collaborative learning. With SCT, personal elements such as expectations, motivation, attitudes, and environmental influences all have a role in learning. SMBCL enhances abilities, self-confidence, and motivation among the students. Al-Rahmi and Zeki (2017) stated that social media-based collaborative learning has significant impacts on student performance, leading to better performance by learners. Social media has become an essential tool for learning for almost one decade. Moghavvemi et al. (2018) investigated that seeking information for entertainment and academic learning usage via social media become a significant source of motivation for learners. Social media usages enter the daily life of students and faculty in this global village-based era. Bozanta and Mardikyan (2017) indicated that social media usage improves students’ interaction with their peers and faculty members. Peer interactions have a significant positive effect on student social media base collaborative learning. Bandura (2009) stated that SCT is a critical theory that explains the synergy of relationships in the literature on collaborative learning, investigating the issue of self-efficacy, collective efficacy, and environmental influences, among others. Westerman et al. (2016) investigated that SMBCL significantly positively affects student and teacher performance, motivating collaborative learning. Balakrishnan and Gan (2016) explored the sentiment that affects students’ different learning styles (i.e., collaborative, participatory, and individual) using social media and found that collaborative learning style has a significant effect on student learning.

Bandura (1997) defines self-efficacy as the idea of one’s ability to perform as expected, so others behave in their own ways and have the confidence to perform successfully in a better way. Prior et al. (2016) said that self-efficacy significantly affects learning management systems and online distance learning environments. Micari and Drane (2011) indicated that students’ self-willingness to participate in group-based learning predicts better academic performance. According to Bandura (2009), self-motivation stems based on evaluating self-investment in one’s activities, and negative and positive responses to their performance. Othman et al. (2012) investigated that collaborative learning enhances the students’ maturity, different experiences, and especially the formation of positive social interactions, such as interpersonal relationships. Chang and Zhu (2012) stated that social media-based collaborative learning experiences influence student involvement and enjoyment through a higher degree of concentration in their studies and social interactions. Alkhathlan and Al-Daraiseh (2017) investigated that social media usage improved collaborative learning, learning process, and engagement of the student in higher education. Henderson et al. (2013) said that social media usage for collaborative learning is beneficial when tasks are well-defined and clear between learner groups. Akçayır and Akçayır (2016) found that 247 Social Sciences Citation Index (SSCI) research papers were published on the use of social network sites up to 2015, and the finding indicated that studies on the usage of social media for educational purposes are increasing every year. These studies significantly affect collaborative student learning, especially in higher education. Choi et al. (2007) stated that the social media-based learning experience improves student learning attitudes and provides more significant outcomes from SMBCL. Braxton et al. (2000) investigated that social media-based collaborative learning influences student attitudes toward active learning. Ituma (2011) discussed that collaborative learning enhances student learning attitudes toward social media-based group learning. Prince (2004) explored that active student learning is distressed by student self-interest and active collaborative learning by their social interactions.

## Hypotheses Development

Based on the literature review, we propose a conceptual model that explains the importance of the following variables in determining learner performance in collaborative learning based on social media: perceived utility, perceived usability, academic self-efficacy, active learning, and student interaction with faculty and learner performance.

### Perceived Benefits

Positive use of social media and social media-based collaborative learning is full of knowledge and information that provides learners with academic and social insight (Pitafi and Ren, 2021). According to Hansen (2006), the interaction between all types of communities has a more significant and positive benefit in exchanging ideas and thoughts. These interactions have positive outcomes and positive perceived benefits. The use of social media and social media-based collaborative learning has positive perceived benefits and a positive attitude toward learning and achieving academic goals, group study and social media-based collaborative learning have perceived positive benefits on student performance and learning in social media-based groups. Based on support from previous studies, our study suggests the following hypothesis:

**H1:** Perceived benefits significantly impact student social media-based collaborative learning.

### Active Learning

According to Garcia and Pintrich (1996), active learning is adjoined with student interest and motivation. Students who participate in social media-based learning perceived positive benefits to academic training and learning (Braxton et al., 2000; Khan et al., 2019). The group-based study has a positive outcome, and students know actively and positively engage with their group mates and mentors via social media-based learning (Sesen and Tarhan, 2010; Pitafi et al., 2018a). Ajaja and Eravwoke (2010) state that social media-based learning has a significant impact on active student learning in closed groups and interactions with their fellows.

**H2:** Active learning significantly impacts student attitudes toward social media-based collaborative learning.

### Perceived Ease of Use

According to previous studies, perceived ease of use of social media has a positive impact on social media-based learning (Pitafi et al., 2020a; Rasheed et al., 2020), and perceived ease of use also has positive relation to student learning and performance toward achievement of goals (Rauniar et al., 2013; Wiid et al., 2013). Perceived ease of use has a positive relationship with the use of social media, and it is also part of the TAM technology acceptance model (Davis, 1989).

**H3:** Perceived ease of use has a significant impact on social media-based collaborative learning of particular university students.

### Perceived Usefulness

As suggested by the technology acceptance model (TAM), the usage of information technology among users strongly depends on its perceived usefulness (Davis, 1989; Venkatesh et al., 2003). In this area, another study performed by Jackson et al. (1997) found no relationship between perceived usefulness and social media-based collaborative learning (Al-Rahmi et al., 2020; Khan et al., 2021). Furthermore, perceived usefulness found a negative association with the use of information technology (Pitafi et al., 2018a). Different researchers also stated no evidence of the perceived usefulness usages relationship. Concluding the above debate, the researchers suggest the following hypotheses.

**H4:** Perceived usefulness significantly relates to social media-based collaborative learning and learner performance.

### Interaction With Student

According to Walker et al. (2008), traditional learning and teaching suffer from limited learning, limited time, and interruptions for both learners and teachers or faculty in the classroom. Draper and Brown (2004) advanced that the medium of technology, interactions, and learning tools break limited class interaction and increase interaction between students and faculty via social media sites in closed or class groups (Pitafi et al., 2020a). This interaction increases and improves positive interactions, and results, and an effective way of learning enhances new education. Blasco-Arcas et al. (2013) stated that inactivity and group-based learning students are highly motivated to participate and complete their goals. In this type of interaction, students’ level of learning and understanding is very high in group learning. Social media-based and technological learning-based groups significantly impact student learning, confidence, and interaction with faculty (Pitafi et al., 2020c). There are more positive and effective learning benefits for shy and/or struggling students, because these type of students more greatly improve their learning and confidence from social media-based collaborative learning (Deil-Amen, 2011).

**H5:** Student interaction with faculty significantly impacts student attitudes toward social media-based learning.

### Social Media-Based Collaborative and Learning Performance

The use of social media increases participation and learning of every segment of society, primarily social media-based collaborative learning, positively impacting student learning, performance, and confidence (Pitafi et al., 2019; Rashid et al., 2020). Online social environments and social websites provide a larger platform for accessible communication to the student for interaction with their mentor and collaborative learning. Ertmer et al. (2011) investigated that by using social media, students completed their assignments and tasks in a good way, and active learning has a positive impact on their collaborative learning. Students with good cognitive ability and skills learn the most from social media-based collaborative learning and complete their tasks in time (Nand et al., 2019). According to Lund (2008), social media-based collaborative learning has a positive impact on student learning ability, and the response of mentors in close social media-based learning groups is very high; therefore, a student gets a timely and rapid response from their fellows and mentor and will complete the final version of their work quickly and publish it on time.

Helou and Rahim (2014) explored the influence of social media sites and the use of social media collaborative learning on student performance. They found that social media sites and collaborative learning significantly positively impact learner performance. There is a significant relationship between cooperative learning, learner performance, and learners’ engagement (Junco, 2012). Hamid et al. (2011) stated that social media in higher education has a positive impact and gives a fruitful result in the education sector.

**H6:** Social media-based learning has a significant impact on learner performance.

### The Moderating Role of Academic Self-Efficacy

Self-efficacy is the ability of a student to complete or fulfill their given task and work in a satisfactory manner (Nand et al., 2019). Micari and Drane (2011) discuss that self-efficacy is an essential element that motivates students to attain their goals, especially in small groups. Self-efficacy decreases social comparison in the sense of lower capability to perform work than their fellows. Self-efficacy increases student confidence, and with higher confidence, students perform better in group tasks and achievements (Nand et al., 2019). High self-efficacy significantly impacts learner performance and motivation to achieve their goals (Pajares, 1996).

**H7:** Academic self-efficacy significantly impacts student social media-based collaborative learning.

## Research Methods

### Research Instruments

Several indicators are used to examine the research model of this study. Previous studies well-known in their respective domains used all of the measurement items. We used a 5-point Likert scale to measure all constructs (Rashid et al., 2022b). Moreover, the 5-point Likert scale was extensively used by several scholars in past studies (Rashid et al., 2022b). The research model of study consists of 11 variables, including control variables. Detail on all the constructs follows (Appendix A).

#### Perceived Benefit

We measure the construct perceived benefit using seven items from Rashid et al. (2022a). The respondents were asked to comment on the perceived benefit of using social media technology as an academic learning tool. The sample question is “Social media prepare me to work in a company.”

#### Active Learning

We measure the construct of Active learning using five items from Molinillo et al. (2018). The respondents were asked to comment on the perceived benefit of using social media technology as an academic learning tool. The sample question is “I learned many factual materials from social media.”

#### Perceived Ease of Use

We measure the construct perceived ease of use using five items from Rauniar et al. (2014) and Sarwar et al. (2019). The respondents were asked to comment on the perceived benefit of using social media technology as an academic learning tool. The sample question is “my interaction with social media/SNS is clear and understandable.”

#### Perceived Usefulness

We measure the construct’s perceived usefulness using seven items from Rauniar et al. (2014) and Sarwar et al. (2019). The respondents were asked to comment on the perceived benefit of using social media technology as an academic learning tool. The sample question is “Using social media/SNS increase my productivity in my coursework.”

#### Interaction With Students

We measure the construct Interaction with students using six items from Barki et al. (2007). The respondents were asked to comment on the perceived benefit of using social media technology as an academic learning tool. The sample question is “I use social media applications to coordinate activities with others.”

#### Social Media Collaboration

We measure the social media collaboration construct using six items from McMillan and Hwang (2002) and Sarwar et al. (2019). The respondents were asked to comment on the perceived benefit of using social media technology as an academic learning tool. The sample question is “I was able to develop my learning abilities through peer collaboration.”

#### Academic Self-Efficacy

We measure the construct of academic self-efficacy using six items from Molinillo et al. (2018). The respondents were asked to comment on the perceived benefit of using social media technology as an academic learning tool. The sample question is “I’m confident I can do an excellent job on the assignments in this collaborative work.”

#### Learning Performance

We measure the construct learning performance using seven items from Ainin et al. (2015) and Sarwar et al. (2019). The respondents were asked to comment on the perceived benefit of using social media technology as an academic learning tool. The sample question is “I feel competent in completing my academic tasks.”

#### Control Variable

We used education, age, and gender as control variables in this study.

### Data Collection Procedure

To achieve the objective of this study, we used a survey procedure to collect data from different universities in China. We used survey methods because the survey method was widely adopted by social science research in past studies (Pitafi et al., 2018b). In addition, the survey method helps to understand the relationship between different variables (Pitafi et al., 2018a). The author conducted this research in China for several reasons. First, Virtual tools are implemented by different Chinese universities to collaborate and communicate with students. Second, China is a country famous for its economy and technology. Third, there are limited studies has been conducted by scholars in the Chinese context to investigate the relationship between social media collaborative platforms and learning performance. The author visited several universities in China to understand students’ use of social media tools. Before conducting the data, the author invited five faculty members and Ph.D. students for review and criticism. After finalizing the questionnaire, the author conducted the pilot study on 50 respondents, and the results were found acceptable. Next, these 50 respondents were removed from the final data set. We included five universities in this survey and collected data from students from different departments. We distributed 700 questionnaires in print format to other classes as all the respondents of this study were fluent in English, so the final questionnaire also used English.

At the start of the survey, we gave out small gifts such as university-printed T-shirts, pens, books, and diaries to students who actively participated in the survey. Within 8 weeks, the author received 625 questionnaires, with a response rate of (89%). We discarded 41 responses during the evaluation, as they were incomplete or filled improperly. Hence, 583 answers were found adequate for this study, and the details of respondents are shown in Table 1.

**TABLE 1:** Demographic information of the samples.

	*N*	Percentage
**Gender**		
Male	224	38.40
Female	360	61.60
**Age**		
21–30 years old	279	47.80
31–40 years old	264	45.20
41–50 years old	41	7.00
**Education level**		
Bachelors/Undergraduate	97	16.60
Masters/Graduate	328	56.20
Doctoral degree	159	27.20

## Data Analysis and Results

### Common Method Variance

Following the guidance of previous studies, the author also conducted the common methods of bias analysis using several procedures. Researchers recommended the CMB test when data was collected from a single source using the same technique (Podsakoff et al., 2003; Kanwal et al., 2020). First, we used the common latent factor method to examine the potential problem of CMB in the current study. Following this procedure (MacKenzie et al., 2011), we looked at the regression weights of all the constructs with and without common factors. Outcomes show that the difference between these two regression weights is no higher than 0.2. Second, we analyzed the correlation of all the variables in Table 2. Results indicated that all the constructs’ co-relation values have values less than 0.90. Third, we used the Herman single factor test on all the variables of all the items; in the resulting total of 45 factors, eight factors have eigenvalues value >1.0 with accountant 72.45%. The first factor has 23.24% of the variance, which is less than 0.50%; therefore, all the results reported that CMV is not a severe issue in this study.

**TABLE 2:** Means, standard deviation, and correlations.

Variable	*M*	SD	1	2	3	4	5	6	7	8	9	10	11
1. Perceived benefit	3.93	0.78	**0.86**										
2. Active learning	4.01	0.36	−0.05	**0.81**									
3. Perceived ease of use	3.98	0.60	−0.04	0.46**	**0.75**								
4. Perceived usefulness	2.95	0.64	−0.11**	0.38**	0.14**	**0.84**							
5. Interaction with students	3.29	0.78	0.00	0.34**	0.28**	0.38**	**0.81**						
6. Social media collaborative	361	0.99	0.09*	0.11**	0.34**	0.01	0.17**	**0.81**					
7. Academy self-efficacy	368	0.81	0.06	0.11**	0.34**	0.06**	0.21**	0.62**	**0.81**				
8. Learning performance	3.62	0.74	0.06	0.29**	0.34**	0.16**	0.10*	0.46**	0.39**	**0.81**			
9. Education level	**NA**	**NA**	0.03	0.03	−0.06	−0.05	0.01	0.00	0.02	0.05	**NA**		
10. Age	**NA**	**NA**	0.05	−0.10*	−0.04	0.08*	0.02	−0.11*	−0.08	−0.17**	−0.21**	**NA**	
11. Gender	**NA**	**NA**	0.01	−0.07	−0.03	−0.07	−0.04	−0.09*	−0.06	−0.10	0.01	0.07	**NA**

*p < 0.05, **p < 0.001. M, mean; SD, standard deviation. The diagonal elements are the square root of the AVE. Boldface numbers are the squirt of the AVE.

### Measurement Model

The fit of the model was assessed using Comparative Fit Index (CFI), Tucker Lewis Fit Index (TLI), and Root Mean Square Error of Approximation (RMSEA) and Chi-square (χ^2^/df) (Hair et al., 2017). Findings shown in Table 3 indicated that the measurement model has the values (TLI = 0.952, CFI = 0.959, NFI = 0.934, IFI = 0.952, REMSA = 0.05, AGFI = 0.902, CMIN/DF = 445.94/174 = 2.49), indicating that all values are less than the cut-off values (Hair et al., 2017).

**TABLE 3:** Comparison measure model and structural model.

Absolute fit measures				Incremental fit measures		Parsimonious fit measures		
Model	*X*^2^/DF	SRMR	RMSEA	NFI	PNFI	CFI	IFI	TLI
MM	4.53	0.07	0.07	0.88	0.76	0.90	0.90	0.89
SEM	1.86	0.05	0.07	0.91	0.89	0.95	0.95	0.95

### Validity and Reliability

The research model of the study has been evaluated using confirmatory factor analysis (CFA), reliability, discernment validity, and convergent validity. Table 4 presented the results of the factor loading, which are greater than the cut-off value of 0.60 (Fornell and Larcker, 1981), of all the constructs’ items. Outcomes of Cronbach’s alpha and composite reliability analysis of all the constructs are also shown in Table 4. Results indicated that values of CR and CA are higher than the recommended value of 0.70 (Fornell and Larcker, 1981; Kanwal et al., 2019). In addition, Table 4 also indicated that the average variance extracted (AVE) scores of all the constructs are higher than 0.50 (Anderson and Gerbing, 1988). These outcomes authenticated that the study model has a satisfactory level of convergent validity.

**TABLE 4:** Results of confirmatory factor analysis.

Variable name	Items	Loadings	CA	CR	AVE
Perceived benefit	PB1	0.852	0.87	0.95	0.74
	PB2	0.871			
	PB3	0.837			
	PB4	0.832			
	PB5	0.899			
	PB6	0.841			
	PB7	0.891			
Active learning	AL1	0.833	0.89	0.91	0.66
	AL2	0.864			
	AL3	0.844			
	AL4	0.773			
	AL5	0.740			
Perceived ease of use	PEOU1	0.690	0.84	0.87	0.57
	PEOU2	0.699			
	PEOU3	0.714			
	PEOU4	0.838			
	PEOU5	0.834			
Perceived usefulness	PUF1	0.757	0.87	0.91	0.64
	PUF2	0.809			
	PUF3	0.830			
	PUF4	0.826			
	PUF5	0.821			
	PUF6	0.757			
Interaction with students	IS1	0.787	0.88	0.93	0.72
	IS2	0.788			
	IS3	0.766			
	IS4	0.874			
	IS5	0.839			
Social media collaborative	SM1	0.913	0.92	0.91	0.66
	SM2	0.808			
	SM3	0.932			
	SM4	0.770			
	SM5	0.749			
	SM6	0.723			
Academy self-efficacy	ASEF1	0.814	0.96	0.93	0.67
	ASEF2	0.846			
	ASEF3	0.898			
	ASEF4	0.778			
	ASEF5	0.755			
	ASEF6	0.829			
Learning performance	LP1	0.860	0.91	0.93	0.66
	LP2	0.804			
	LP3	0.812			
	LP4	0.898			
	LP5	0.763			
	LP6	0.678			
	LP7	0.860			

*Items, no of items used in constructs; Loadings, Factor Loading; CA, Cronbach’s alpha; CR, composite reliability; AVE, average variance extracted.*

Moreover, we also analyzed the decrement validity of the study model using the outcomes in Table 2. The findings from Table 2 indicated that the AVE square root of all the constructs is higher than the inter-correlation values of all the constructs, which conforms to the decrement validity of the research model (Fornell and Larcker, 1981). In summary, the results of Tables 2, 4, authenticated that the study model has a satisfactory level of decrement validity.

### Structural Model

The proposed study model was examined using a structural equation modeling (Hair et al., 2017). The outcomes shown (TLI = 0.941, CFI = 0.948, IFI = 0.948, AGFI = 0.894, NFI = 0.911, CMIN/DF = 612.60/268 = 2.28, REMSA = 0.05) reveal that all the values are above minimum level (Hair et al., 2011).

**FIGURE 1:**
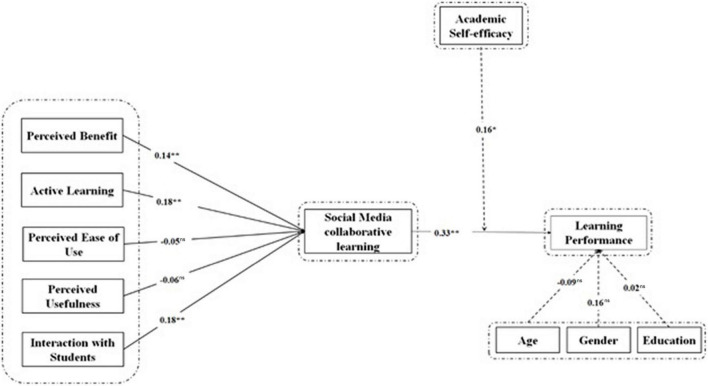
Structural model. *p < 0.05, **p < 0.001. ns, non-significant.

Figure 1 indicates the results of structural equation modeling of research model. Results indicated that perceived benefit (β = 0.14, *t* = 2.61, *p* < 0.001), active learning (β = 0.18, *t* = 2.75, *p* < 0.001), perceived ease of use (β = −0.05, *t* = −1.14, *p* < 0.001), perceived usefulness (β = −0.06, *t* = −1.62, *p* < 0.001), and interaction with students (β = 0.18, *p* > 0.05) have significant relationships with social media collaborative. H1, H2, H3, H4, and H5 are therefore validated by the current data set. In addition, social media collaborative learning also has significant effect on learning performance with (β = 0.33, *t* = 4.85, *p* < 0.001), meaning H6 is also validated by this study.

### Moderation Analysis

To analyze the moderation effect of academic self-efficacy, we used hierarchical regression analysis, as shown in Table 5. The findings of Table 5 indicated that the dependent variable learning performance regressed in four steps. In step 1, we first entered the control variable, as shown in Table 5. In step 2, we entered social media collaboration as an independent variable; results indicated that social media collaborative learning significantly affects learning performance with (β = 0.44, *p* < 0.001). In step 3, academic self-efficacy was entered, and the results validated that academic self-efficacy significantly affects learning performance (β = 0.16, *p* < 0.05). Finally, we entered the interaction terms (Social media collaborative × Academic self-efficacy), and the results were also significant (β = 0.18, *p* < 0.05), thereby supporting H7. Therefore academy self-efficacy positively moderates the association between collaborative learning and learning performance.

**TABLE 5:** Results of hierarchical regression.

Regression analysis				

Variable	Model 1	Model 2	Model 3	Model 4
Gender	−0.09	−0.05	−0.05	−0.04
Age	0.16	0.10*	−0.10*	−0.12*
Education	0.02	0.03	−0.02	0.02
**Main effects**				
Social media collaborative		0.44**	0.34**	0.34**
**Moderator**				
Academy self-efficacy			0.16*	0.25**
**Interactions**				
Social media collaborative × Academy self-efficacy				0.18**
*R* ^2^	0.03	0.23	0.0.24	0.27
Adjusted *R*^2^	0.03	0.22	0.024	0.26
Changed *R*^2^	0.03	0.19	0.01	0.02
F Change	7.89	146.0	12.08	20.05

*p < 0.05, **p < 0.001.

**FIGURE 2:**
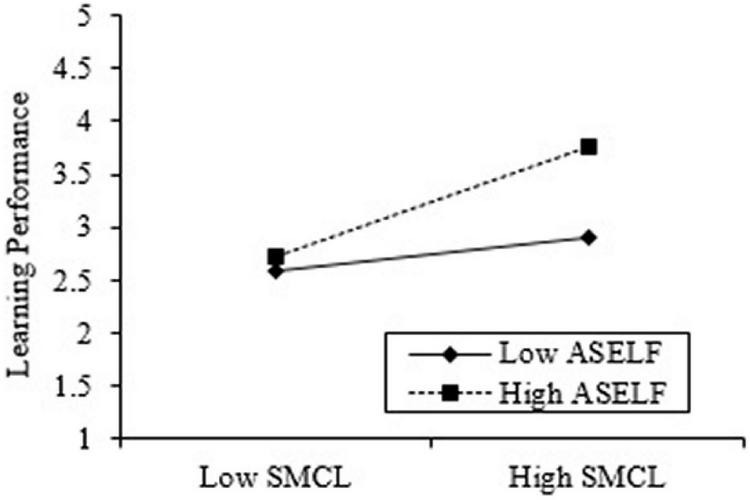
Moderating effect of academic self-efficacy with the relationship of social media learning collaborative and learning performance. SMCL, social media learning collaborative; ASELF, Academic Self-efficacy.

Furthermore, to understand the moderating effect of academy self-efficacy, we employed graphical procedures (Cao et al., 2020), as shown in Figure 2. Figure 2 presents the regression line of moderating influence of academy self-efficacy with the link between social media collaborative learning and learning performance.

## Discussion, Implication, Limitation

### Discussion

This research aimed to look into some of the factors that influence students’ attitudes about collaborative learning based on social media. The empirical research backs up the recommended study model.

The findings indicate that three characteristics are significant predictors of attitudes toward flow experience, active and collaborative learning, and perceived advantages. First, this study reveals that active learning is a fundamental component required for a favorable attitude toward collaborative effort because the individuals involved work out their specific learning, which is consistent with previous research such as Garcia and Pintrich (1996). Also, active learning has a good impact on collaborative learning. This finding is similar to prior research, which discovered that active learning is directly associated with involvement and people’s views toward various online actions, generating interest in the business, educational, and scientific domains. Further, it also confirms the premise that when students see the assistance given to scholarly activity, their approach toward collaborative learning develops, focusing on completing the course and gaining skills and knowledge (Jones et al., 2012).

Moreover, Micari and Drane (2011) indicate that students with high academic self-efficacy are less concerned by social comparisons because they are more confident in their abilities. In a collaborative work environment, students who are more interested in equating themselves to other group members may experience less flow. This could be because they lack confidence in their actions and, as a result, reduce their engagement with their peers, feeling less invested in the task (Nand et al., 2019).

These outcomes are also in line with earlier studies (Hwang et al., 2015) demonstrating that active learning, perceived simplicity of use, perceived usefulness, and engagement with students had a favorable influence on social media cooperation. Active learning in a collaborative work atmosphere promotes a more incredible penchant for social media collaboration. Students’ enhanced attentiveness and enthusiasm improve their integration with classmates, inspiring them to become more participative. It has also been demonstrated that students who are more interested in learning are more tolerant of active learning, explaining the significance of students’ interest in the topic curriculum and accompanying activities (Schoor et al., 2011). In the same way, students embrace active learning more readily when they feel better connected with their classmates in a group with whom they share common aims and a sense of belonging (Skalicky et al., 2013). Results indicate that perceived ease of use and perceived usefulness have an insignificant impact on collaborative learning, both hypotheses are rejected by this study. The possible reason for this rejection is that social media applications are mostly used by students for enjoyment and communication purposes. Future scholars are suggested to conduct depth study and highlighted the possible advantages of social media applications.

Accordingly, this study adds to the understanding of collaborative learning from the perspective of social media by emphasizing the importance of attitude. In this respect, this article adds to the body of knowledge about attitudes toward learning by examining academic self-efficacy as a moderator between social media collaboration and learning performance. This study also shows how this variable influences collaborative and active learning attitudes. Likewise, this study backs dynamic learning approaches by emphasizing their significance in fostering good attitudes toward collaborative learning (Nand et al., 2019).

### Theoretical Implications

This study also has several contributions. First, in this study, we highlighted the role of social media as a collaborative tool. Previous studies have investigated the role of social media on academic performance by investigating the factors of social media applications (Nand et al., 2019). Second, this study extends the existing literature on social media by investigating the significant impact of social media collaborative learning on student academic performance. From this perspective, this study theoretically and practically examined the factors that affect student academic performance. Third, the findings of this research also help the students and educational institutes the usage of social media for learning purposes. To benefit from social media for learning purposes universities/colleges may craft awareness among students/faculty members by conducting seminars to explain the advantages of social media for learning performance. Even a small significant use of social media can help the students to enhance their academic performance.

Finally, social media applications should be adopted in such a way that they may incorporate teaching materials with several interactions, knowledge sharing, and interesting exercises to enable the learning process. For example, using social media and creating online tasks may be a better way to engage students in the learning process.

### Practical Implications

Based on the results, suggestions can be developed for faculty members and students by utilizing social media platforms to improve the outcomes of these activities. To begin with, given that, regardless of enhanced interaction amongst group participants, their flow is still lowered, this study backs up other researchers’ advice that teachers must form a happy atmosphere (Kwon et al., 2014), along with defining and planning the imperatives of action (Pitafi et al., 2020b). First, teachers should communicate all errands, the group assessment system (Rashid et al., 2020) and measures from the very start of the process; thereby encouraging communication mechanisms, for instance, the use of humor (Rashid et al., 2019). Second, teachers must thoroughly comprehend the aspects that boost student engagement to develop the flow experience, allowing students to focus and delight in collaborative work (Jiang et al., 2019).

Third, students will be more inclined to participate in active learning if group work is well-managed, allowing teachers to offer new approaches and activities that enable students to accomplish their learning processes. Furthermore, the task objectives and learning advantages must be communicated clearly beyond getting a good grade. Students’ attitudes and flow experience will be reinforced if they are devoted to their peers, comprehend the benefits of collaborative work, and have a propensity toward active learning (Jones et al., 2012).

### Limitations

As with other studies, this study has few limitations, which may provide prospects for a prospective further study. First, we checked and suggested the usage of social media in the collaborative learning process among university students in China; future studies can further test our idea in diverse countries with diverse cultures to obtain more outcomes. Second, the measurement scales were obtained from an earlier study and amended, and therefore there might have been some misspecification of the variables. Lastly, our research is limited to collaborative learning among university students using social media. Future studies could examine actual shopping behavior and cogitate other concepts that might describe the variances between intentions and behavior.

## Conclusion

This study investigates the use of social media in the collaborative learning process among Chinese university students. Various aspects of improving collaborative learning among university students in the context of using social media will be investigated in this study. Results show that perceived benefit, active learning, and interaction with students are significantly related to social media collaboration. However, perceived ease of use and usefulness has an insignificant effect on social media collaborative learning. While students’ academic self-efficacy significantly moderates the relationship between social media collaboration and learning performance.

## Data Availability Statement

The original contributions presented in the study are included in the article/supplementary materials, further inquiries can be directed to the corresponding author/s.

## Ethics Statement

Ethical review and approval was not required for the study on human participants in accordance with the local legislation and institutional requirements. Written informed consent from the patients/participants was not required to participate in this study in accordance with the national legislation and the institutional requirements.

## Author Contributions

All authors listed have made a substantial, direct, and intellectual contribution to the work, and approved it for publication.

## Conflict of Interest

The authors declare that the research was conducted in the absence of any commercial or financial relationships that could be construed as a potential conflict of interest.

## Publisher’s Note

All claims expressed in this article are solely those of the authors and do not necessarily represent those of their affiliated organizations, or those of the publisher, the editors and the reviewers. Any product that may be evaluated in this article, or claim that may be made by its manufacturer, is not guaranteed or endorsed by the publisher.

## Appendix

**APPENDIX A:** Survey questionnaire.

Constructs and measurement	Scale
**Gender** (I) Male (II) Female	
**Age** (I) 21–30 Years old (II) 31–40 Years old (III) 41–50 Years old	
**Education** (I) Bachelors/Undergraduate (II) Masters/Graduate (III) Doctoral Degree	
**Perceived Benefit** 1 Learn about group dynamics. 2 Increased ability to effectively work in a group. 3 Improved communication skills. 4 Prepare me to work in a company. 5 Demonstrated division of labor. 6 Simulates what I will experience in the work world. 7 Have made new friends (item dropped).	Likert 1–5
**Active Learning** 1 Learned many factual materials. 2 Identified central issues in the field. 3 Became more interested in the subject. 4 Participated actively. 5 Written assignments aided the student’s learning.	1–5 Scale
**Perceived Ease of Use-5** 1 My interaction with social media/SNS is clear and understandable. 2 Social media is flexible to interact with it. 3 It is easy to become skillful at using social media. 4 I do not have any problem learning about social media sites’ features.	1–5 Scale
**Perceived Usefulness-6** 1 Using social media/SNS increases my productivity in my coursework. 2 Social media/SNS is used to communicate with more people in a short period. 3 Using social media enables me to improve my learning performance.	1–5 Scale
**Interaction with students-6** I use social media applications to solve various problems. I use social media applications to exchange knowledge with other people. I use social media applications to plan or follow up on my tasks. I use social media applications to coordinate activities with others. I use social media applications to interact with customers. For accomplishing my tasks, social media application is essential.	Likert 1–5 Scale
**Social media collaborative-6** 1 I was able to develop my learning abilities through peer collaboration. 2 I was able to develop new knowledge and skills from other members of social media. 3 I was able to develop a more comprehensive understanding of the topics through group discussion.	Likert 1–5 Scale
**Academy self-efficacy** 1. I’m confident I can do an excellent job on the assignments in this collaborative work. 2. I’m certain I can understand the most difficult material presented in the readings for this collaborative work. 3. I’m confident I can learn the basic concepts taught in this collaborative work. 4. I expect to do well in this collaborative work. 5. I’m certain I can master the skills being taught in this collaborative work. 6. I’m confident I can understand the most complex material presented by the instructor in this collaborative work. 7. I believe I will receive an excellent grade in this collaborative work. Considering the difficulty of this collaborative work, the teacher, and my skills, I think I will do well in this class.	Likert 1–5 Scale
**Learning Performance-7** 1 I feel competent in completing my academic tasks. 2 I have learned how to do my task compilation efficiently. 3 I have performed academically as well as I expected I would.	Likert 1–5 Scale
